# Locomotion Induces Fundamentally Different Patterns of Ca^2+^ Signaling in Astrocytes and Neurons

**DOI:** 10.1093/function/zqad028

**Published:** 2023-06-05

**Authors:** Hajime Hirase, Maiken Nedergaard

**Affiliations:** Center for Translational Neuromedicine, University of Copenhagen, Copenhagen 2200, Denmark; Center for Translational Neuromedicine, Department of Neurosurgery, University of Rochester Medical Center, Rochester, NY 14642, USA; Center for Translational Neuromedicine, University of Copenhagen, Copenhagen 2200, Denmark; Center for Translational Neuromedicine, Department of Neurosurgery, University of Rochester Medical Center, Rochester, NY 14642, USA

## A Perspective on “Dissociation Between Neuronal and Astrocytic Calcium Activity in Response to Locomotion in Mice”

A modest literature links the onset of locomotion with increased intracellular calcium (Ca^2+^) in neurons and astrocytes of the sensory and motor cortex, but a formal comparison of Ca^2+^ responses in the two cell types has been lacking. The new study by Fedotova et al.^1^ systematically compared Ca^2+^ activity induced by locomotion in neurons and astrocytes of awake, behaving mice. A main strength of this study lies in the detailed analysis of Ca^2+^ dynamics for the two cell types, in consideration of the coupled activity of neurons and astrocytes during spontaneous locomotion. The authors used recombinant adeno-associated viral (AAV) vectors to express the genetically encoded Ca^2+^ sensor GCaMP6f selectively in either astrocytes or excitatory neurons (AAV5-gfaABC1D-cyto-GCaMP6f or AAV2/9-CamKII-GCaMP6f, respectively) in the somatosensory cortex. The mice were first trained to accommodate head-fixation to a mobile home cage device mounted on a microscope stage, with detection of locomotion onset by tracking software, thus enabling an accurate temporal correlation of locomotion onset with Ca^2+^ dynamics. The authors found that astrocytic Ca^2+^ rose upon initiation of movement, with first appearance in the distal processes, followed by increases of magnitude in the cell bodies. Unlike the astrocytes, neurons displayed Ca^2+^ activity during rest, with more rapid onset of Ca^2+^ elevation by locomotion. Thus, neurons and astrocytes show distinct temporal/spatial dynamics of intracellular Ca^2+^ during the initiation of voluntary movement.

**Figure 1.**
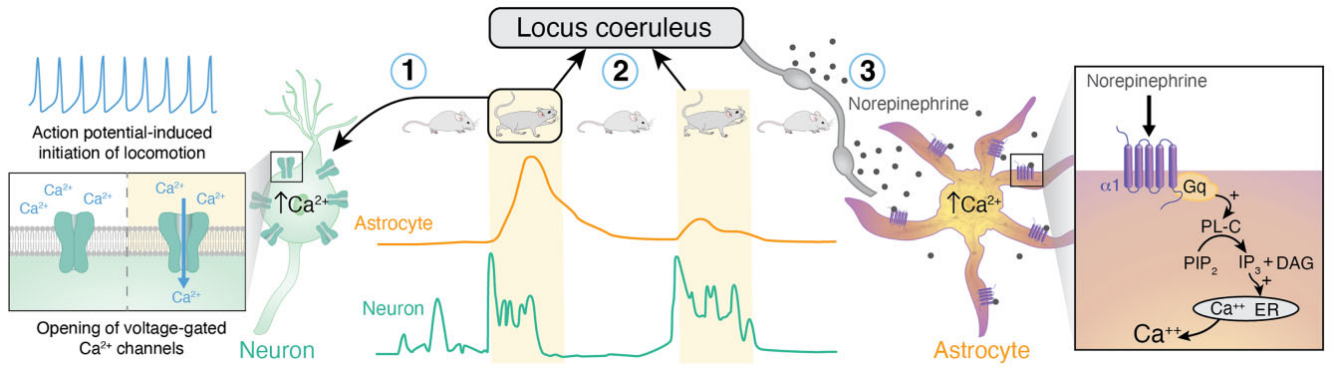
A schematic illustration of locomotion-associated principal Ca^2+^ dynamics in neurons and astrocytes. Sensory and motor activation induces action potentials in neurons in the somatosensory cortex. The fast neuronal Ca^2+^ increases are primarily driven by activation of voltage gated Ca^2+^ channels, which induces the rapid influx of Ca^2+^ from the extracellular space (1). The norepinephrinergic locus coeruleus is activated at the onset of locomotion, which gives rise to volume transmission of NE in the cortex (2). NE activates astrocytic alpha-1 adrenergic receptors, which in turn induce intracellular Ca^2+^ mobilization via the Gq pathway (3). The sequential Gq-pathway provokes a relatively slow and delayed mobilization of Ca^2+^. Fedotova et al. now report that astrocytic Ca^2+^ signaling is suppressed or absent upon a repeated locomotion episode. Multiple mechanisms might contribute to the decrease in astrocytic Ca^2+^ responses to locomotion.

Perhaps the most novel part of the study is in the comparison of Ca^2+^ responses to repeated episodes of locomotion (Figure 1). In that analysis, neurons exhibited a stereotypic increase in Ca^2+^ with each episode, whereas astrocytes showed dampening of successive responses. What are we to make of this intriguing observation? First, we note that neuronal Ca^2+^ signaling relies mainly on voltage-gated Ca^2+^ channels in the plasma membrane that are activated at each action potential. In mammals, locomotion is ultimately initiated by brain stem activation that integrates higher-order input from the basal ganglia and cortex.^2,3^ Locomotion-associated neuronal Ca^2+^ activities in the somatosensory cortex are likely induced by action potential discharges involved in somatosensation. By contrast, Ca^2+^ signaling in astrocytes relies on mobilization of intracellular Ca^2+^ stores, which is inherently a much slower pathway as it must go through several biochemical steps from receptor activation to the opening of IP_3_ receptor-activated Ca^2+^ channels in the endoplasmic reticulum to release internal Ca^2+^ stores. Norepinephrine (NE) release from locus coeruleus projections is the initiator for astrocytic cytoplasmic Ca^2+^ increases upon locomotion; agonism at alpha-1 adrenergic receptors leads to the delayed mobilization of intra-astrocytic Ca^2+^ stores. Conversely, alpha-1 receptor antagonists such as prazosin block not only locomotion-induced astrocytic Ca^2+^ increases, but also >90% of spontaneous astrocytic Ca^2+^ events, thus positing norepinephrinergic (noradrenergic) transmission as the primary driver of cortical astrocytic Ca^2+^ signaling.^4^ The alpha-1 receptor is a G_q_-linked G protein-coupled receptor (GPCR) with abundant expression in astrocytes and cortical neurons,^5^ although alpha-1 receptors induce large-amplitude cytosolic Ca^2+^ surges predominantly in astrocytes.^6^

The authors interpreted their observation of attenuated astrocytic Ca^2+^ response upon contingent locomotor initiation as indicating a refractory period due to Ca^2+^ mobilization from the endoplasmic reticulum. We note that GPCR activation also entails a refractory period that is determined by a desensitization/re-sensitization mechanism involving the heterotrimeric G protein complex.^7,8^ Upon GPCR activation, the G_α_ and G_βγ_ G protein complex subunits are detached from the membrane-bound receptor. G_α_ initiates the downstream signaling cascade, whereas G_βγ_ promotes the recruitment of GPCR kinases (GRKs) that phosphorylate the C-terminal tail of the GPCR to prevent G protein binding. The phosphatase PP2A eventually dephosphorylates the C-terminal, thus reinstating the responsiveness of the GPCR. Moreover, a recent study demonstrated that distinct GRKs bind to G_αq_, the initiator of the G_q_ pathway, to drive rapid desensitization.^9^ Regardless of the various mechanisms, minute-scale refractory periods of locomotion-induced astrocytic Ca^2+^ signaling are a signature of GPCR activation. For instance, repeated activation of the optogenetic GPCR Optoα1AR shows a similarly attenuated response between the first and the second activations at an interval of 60 s.^10^

The authors proposed that the neuronal Ca^2+^ increases are involved in the initiation and command of the skeletal muscle activity. On the other hand, in their analysis, the astrocytic Ca^2+^ responses were clearly delayed, first appearing seconds after the onset of locomotion. Prior studies have suggested that the delayed astrocytic Ca^2+^ increases upon locomotion promote local blood flow, energy metabolism, K+ buffering, and the termination of neuronal signaling. Now that genetically encoded fluorescent biosensors and opto-/chemo-genetic actuators have come of age, we anticipate that future studies shall address the functional consequence of astrocytic Ca^2+^ signaling at various spatio-temporal scales. The detailed analysis now presented in Fedotova et al. brings us a step closer to deciphering the fundamentally different types of signaling in astrocytes and cortical neurons, and how each cell type contributes to optimizing motor-related brain activity such as the initiation of voluntary locomotion.
